# Clinical Adoption of mHealth Technology to Support Pediatric Cystic Fibrosis Care in Sweden: Qualitative Case Study

**DOI:** 10.2196/11080

**Published:** 2018-12-10

**Authors:** Meghan Longacre, Stuart Grande, Andreas Hager, Meera Montan, Rikard Palmer Bergquist, Maria Martensson, Greg Kotzbauer

**Affiliations:** 1 The Dartmouth Institute for Health Policy and Clinical Practice The Geisel School of Medicine at Dartmouth Dartmouth College Lebanon, NH United States; 2 Upstream Dream Bromma Sweden; 3 Lund Pediatric Cystic Fibrosis Center Skane University Hospital Lund Sweden

**Keywords:** cystic fibrosis, mHealth, mobile phone, pediatrics, qualitative case study, technology

## Abstract

**Background:**

Mobile health (mHealth) technologies have potential to improve self-management and care co-ordination of pediatric chronic diseases requiring complex care, such as cystic fibrosis (CF). Barriers to implementation include the lack of support and infrastructure to use mHealth in the clinical microsystem. Coproducing mHealth technology with patients, clinicians, and designers may increase the likelihood of successful integration into the clinical setting.

**Objective:**

This study explored the development, adoption, and integration of a new, co-produced mHealth platform (Genia) for the management of pediatric CF in Sweden.

**Methods:**

A retrospective, qualitative case study approach was used. The case was defined as the process of introducing and using Genia at the Pediatric Cystic Fibrosis Center at Skåne University Hospital in Lund, Sweden. Data sources included interviews, presentations, meeting notes, and other archival documents created between 2014 and 2017. To be included, data sources must have described or reflected upon the Genia adoption process. Iterative content analysis of data source materials was conducted by 2 qualitatively trained researchers to derive themes characterizing the mHealth clinical adoption process.

**Results:**

In total, 4 core themes characterized successful clinical integration of Genia in Lund: cultural readiness to use mHealth; use of weekly huddles to foster momentum and rapid iteration; engagement in incremental “Genia Talk” to motivate patient adoption; and co-design approach toward pediatric chronic care.

**Conclusions:**

Principles of quality improvement, relational co-ordination, user-centered design, and coproduction can facilitate the integration of mHealth technology into clinical care systems for pediatric CF care.

## Introduction

Longitudinal care of children diagnosed with cystic fibrosis (CF)—an autosomal recessive genetic disorder affecting lung capacity—is characterized by substantial personal, familial, and medical burden [1,2]. To decelerate decline in lung function, children must engage in respiratory and physical therapies up to 2 hours per day, adherence to which often decreases as children age through adolescence [3]. Guidance from health care providers is frequently disconnected from this daily routine and the personal goals of pediatric CF patients [4]. For pediatric CF, this disconnectedness derives in part from the traditional model of episodic care delivery, which hinders an uninterrupted approach and may result in fragmented clinical care [5]. The challenge to co-ordinated care particularly impacts young people during a developmentally vulnerable time, when they move from clinician-regulated pediatric care to increasingly autonomous self-management of their illness [6]. Successful transition to adult CF care is dependent on consistent, appropriate, and increasingly independent maintenance of a care regimen [7]. Encouraging these patterns of behavior in late childhood and early adolescence fosters stability and reliability of personal management of chronic illness into adulthood [8].

Mobile health (mHealth) technology-based platforms offer a transformational mechanism for improving clinical care in both preventive medicine and chronic disease management [9,10]. One strength of mHealth as a disease management tool is its ability to leverage existing mobile technology infrastructure and the ubiquity of smartphones across populations [11]. For adolescents, who are frequent users of mobile technology, mHealth applications show potential as a strategy for improving self-management of and adherence to treatment regimens for numerous chronic conditions [12]. Successful use of technology-based support systems to foster self-management, however, is not without challenges. Usability studies identify barriers to the uptake of and adherence to various mHealth and electronic health systems used by clinicians and patients [13,14]. These barriers include low self-efficacy with mHealth platforms; perceptions that the technology will not enhance clinical outcomes; privacy and security concerns; and lack of infrastructure to support the use of mHealth applications [15,16].

Integral involvement of end users in the development of mHealth applications—such that the technology is “co-produced” by designers, clinicians, and patients—may be a strategy to overcome implementation challenges. Coproduction refers to the joint creation of health care services for managing the treatment of a health condition [17]. By engaging end users in mHealth development, this joint design approach may avoid common implementation barriers and enhance the likelihood that these new technologies will be adopted in clinical settings and used by target audiences [18,19].

The purpose of this study was to provide a retrospective case analysis of the development, adoption, and integration of a new, co-produced mHealth platform for the management of pediatric CF in Sweden. Genia is an app-based patient support system (PSS) designed to foster collaborative care and enhance self-management among pediatric patients living with chronic conditions. In 2014, Genia was introduced to the Pediatric Cystic Fibrosis Center at Skåne University Hospital (Lund, Sweden) in its design phase and codeveloped with adolescent patients and CF providers at the center. The adoption of Genia in Lund was highly successful, with a majority of pediatric CF patients using the app. The aim of this case study was to examine the introduction of Genia in Lund to identify successful contributors to the clinical integration of a technology-based PSS, which may be disseminated to other pediatric CF centers in Sweden and abroad and potentially to other pediatric chronic conditions.

## Methods

### Mobile Health Patient Support System: Genia

**Figure 1:**
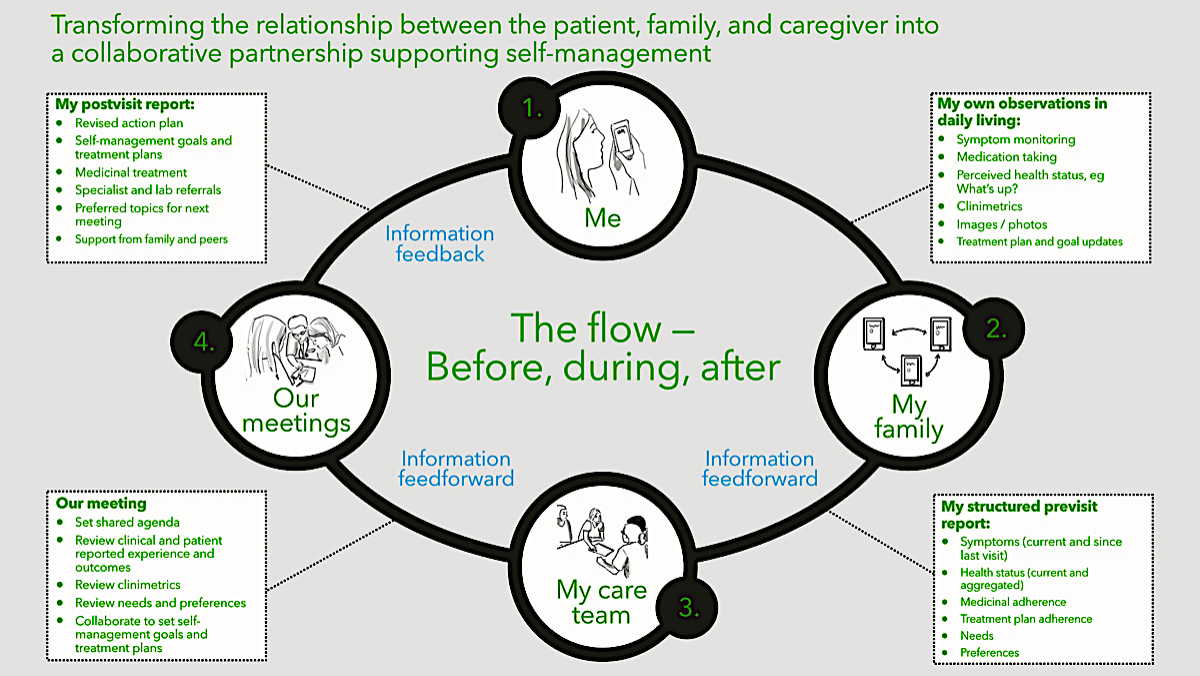
Genia flow.

Genia is a mobile iOS PSS created by a Swedish-based company Upstream Dream to optimize consensus-building in pediatric care by improving communication between patients and clinical teams, fostering disease self-management and aligning patients’ goals with clinical treatment plans (see Figure 1).

**Figure 2:**
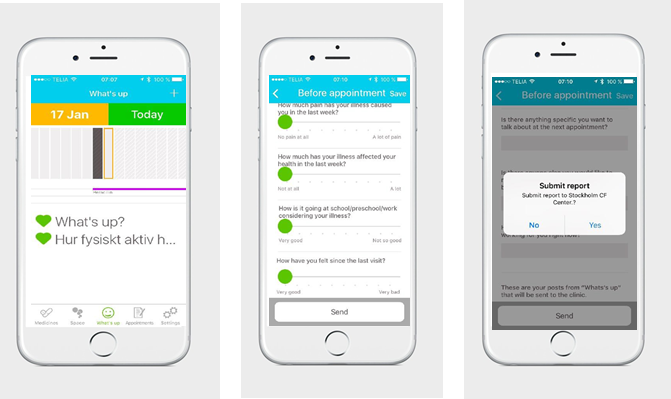
Screenshots of sample Genia app pages developed Upstream Dream.

By doing so, Genia aims to facilitate timely, meaningful, and appropriate clinical care and ultimately to improve patients’ quality of life. Through Genia*,* patients (or parents, depending on the patient’s age) can record daily health observations and symptoms between visits (eg, physical activity or gastrointestinal problems), track medications, and complete previsit reports, including treatment preferences and goals, prior to a clinical appointment. This patient-reported information allows patients to document their disease activity and preferences in the real-time between clinical visits (see Figure 2).

Patient data are then integrated into the National CF Quality Registry (ie, a registry established in 1992, encompassing all 21 regional health care systems or payors in Sweden, which longitudinally follows every CF patient in Sweden) and the care flow within the clinical setting. Clinical providers—including physicians, physiotherapists, and others—are able to review patients’ previsit reports as an Adobe PDF file in the CF registry prior to the clinical visit to better inform the visit and foster opportunities for shared decision making and goal setting. Patients and providers also use Genia to collaboratively document agreed upon therapeutic decisions, actionable steps, and other information derived during the clinical visit. Genia thus aims to foster patient self-management, build trusted patient-provider relationships, and increase compliance with mutually agreed upon care plans.

Grounded within principles of user-based design [20], feedforward systems [21], and coproduction [17], functional features of Genia were created and tested through small-scale, iterative design cycles with patient and family representatives and clinical CF providers within the Lund Pediatric CF clinical microsystem. These learning collaboratives formed the backbone of Genia development by ensuring that technological modifications of the app were grounded in end user’s lived experiences. This user-centered perspective is also illustrated in the key features of the app, which identify patients as the experts in their disease state and better enable them to provide their voice to the care they receive. Genia is currently available for download from the iTunes App Store in an iOS platform in Swedish and English.

### Study Design

We conducted a retrospective, descriptive, qualitative case study [22] of Genia adoption in Lund as part of a larger formative evaluation of Genia within the clinical pediatric CF setting in Sweden. A qualitative approach was selected to allow for an in-depth, inductive examination of data within a naturalistic setting (ie, Lund), enhancing the validity of findings. The case study method was considered most appropriate for several reasons, including its holistic approach to describing how and why Lund was successful; the emphasis on exploration of a phenomenon in the “real-life” clinical settings where the researchers observe but do not manipulate behaviors or processes under examination; and the ability to conduct a retrospective analysis by examining historical artifacts documenting the Genia development and adoption processes [22]. This study was reviewed and determined to be exempt by the Committee for the Protection of Human Subjects at Dartmouth.

### Study Setting

The “case” was defined as the process of introducing and using Genia at the Pediatric Cystic Fibrosis Center at Skåne University Hospital. Located in Lund, Sweden, Skåne University Hospital represents 1 of only 4 pediatric hospitals in Sweden. The Lund CF Center is a regional center for specialized CF care in accordance with the European Cystic Fibrosis Society Standards of Care and serves approximately 60 pediatric patients annually. The pediatric clinical team in Lund consists of 4 physicians, 2 nurses, 2 physiotherapists (ie, physical therapists), 1 dietician, 1 social worker, and 1 psychologist. This team meets weekly to discuss care plans and clinical issues with incoming patients. Pediatric CF patients located geographically close to the center typically visit the clinic every 2 months. Those living outside the region visit with the Lund clinical staff 2-4 times per year. Lund has a long tradition of emphasizing the role of physiotherapy for the care of pediatric CF patients. Reflecting this tradition, at each clinical appointment, physiotherapists have an hour-long appointment with the patient prior to the physician’s visit with the patient.

Genia was initially introduced to the Lund CF Center in late 2014. By July 2017, the Lund CF clinical staff indicated successful integration of Genia into their practice, defined as the percent of patient Genia users and Genia use within care team meetings. Specifically, 87% of Lund CF pediatric patients were members of the Genia PSS, and 85% of all care team meetings (ie, clinical visits between pediatric CF patients and care providers) were supported by Genia through the use of a previsit report.

### Data Sources

Consistent with the case study methodological approach, we employed a data triangulation strategy in which data were extracted from multiple sources that were created between late 2014 and July 2017. Data sources included interviews with Lund providers and the Genia design team, design and implementation meeting notes, design team and clinical team emails, archived presentations, Genia progress reports, and other documents. To be included in the analysis, data sources must have described or reflected upon the early adoption and integration process of Genia in Lund. Some archival data sources were translated from Swedish to English using Google translate or by a bilingual member of the Genia design team. All data sources were organized and condensed into a tabular case record in Microsoft Excel in preparation for analysis [22].

### Data Analysis

Data analysis was conducted by 2 qualitatively trained researchers unaffiliated to either the Genia design team or the pediatric Lund CF team. We applied iterative, conventional content analysis [23] in the coding process. Conventional content analysis is an appropriate approach when there is not a strong theoretical framework directing the analysis, the aim of the investigation is descriptive, and study design is primarily observational [23]. Because of this, our analytic approach was primarily inductive, in which we allowed themes to emerge from the raw data. We identified themes within 3 temporal stages of Genia adoption: (1) PSS introduction to the CF team, (2) facilitators of widespread adoption, and (3) integration into clinical flow.

The first researcher conducted the initial data analysis using the aggregate case record as the full data source. Following immersive review of the data, an initial round of preliminary coding was performed for the entire case record [24]. Due to the varied types of data sources, we employed multiple coding techniques, including descriptive coding to document the data source and timeline and process coding to identify the strategic actions during Genia adoption and implementation [24]. When possible, we also used direct words or phrases from the data source as emergent, *in vivo* codes. During the second round of coding, we looked for patterns of codes within and across the temporal stages of Genia adoption, expanding, synthesizing, reframing, or rephrasing codes as necessary. Larger themes were described and illustrated with exemplars. The second researcher reviewed the case record as well as the thematic and illustrative evidence. Clarifications and discrepancies were resolved through consensus discussion. To enhance the credibility of findings, the final set of themes was provided for review to 1 member of the pediatric Lund CF team and 2 members of the Genia design team. No changes were made after their review.

## Results

### Principal Results

In total, 4 core themes characterized the successful adoption and integration of Genia in Lund. Illustrative quotes are excerpted from interviews with pediatric Lund CF providers.

### Cultural Readiness to Use Mobile Health

Attitudinal and structural characteristics of Lund contributed to a pre-existing culture that was receptive to the introduction of a new mHealth technology. The Lund provider team had undertaken informal quality improvement (QI) projects prior to Genia and were engaged and motivated to continue a QI-type approach to their clinical work.

I think we always have that in mind to work ‘Lean’ [Lean Six Sigma] and to make quality improvement better at the clinic.

Additionally, Lund providers indicated that Genia represented a more “modern” way of engaging in their clinical work with pediatric CF patients, particularly due to its technology-based platform, and thus it was consistent with their attitudinal ethos of continual improvement.

Several structural characteristics of Lund also contributed to the success with which the clinic adopted Genia. The historical prominence of the physiotherapist in pediatric CF care management yielded a trusted, internal point person to manage the effort of introducing Genia. The lead physiotherapist in Lund served as the primary “champion” of Genia and was the person who initially familiarized the wider clinical team to the app. Because of her role, she was influential in the decision by other members of the clinical team to try Genia.

It’s a tradition in Sweden and especially in Lund that the physio[therapist] is very important and mostly everyone listens to the physio. So if the physio says something, I really believe that everyone is listening.

The second physiotherapist in Lund supported the lead physiotherapist’s efforts and likewise served as a Genia champion. The presence of 2 clinical champions at the site allowed for a collaborative approach and shared responsibility for the workload associated with Genia onboarding.

You have to be at least two to get this project working because it’s hard to do everything by yourself. You need two fighting spirits.

The physiotherapists’ historical leadership role in pediatric CF care helped not only with onboarding other clinical providers to Genia but also with onboarding patients. The physiotherapists represent the most familiar clinic-based providers to the patients due to the extended time they spend with patients (ie, 1 hour) prior to each appointment. The close, trusted nature of the physiotherapist-patient relationship supported the physiotherapists’ ability to introduce Genia to the Lund pediatric patients.

Finally, because the Lund CF clinic is relatively small (~60 patients) and the pediatric CF provider team is also small (ie, 11 members), logistical co-ordination and communication regarding Genia was easier. The team already met weekly to discuss patient issues; thus, integrating Genia into these team meetings was a “natural” way to incorporate Genia into a regular clinical discussion.

### Weekly Huddles Fostered Forward Momentum and Rapid Iteration

Short (ie, 15 minute) weekly clinic “huddles” served as the critical process mechanism for fostering continued, forward momentum in adopting Genia in Lund. Huddles were facilitated by a Genia design team member and attended by the physiotherapist champions, other clinical team members, and adolescent CF patient “lead users.” Huddles offered opportunities to provide support for using Genia, understand barriers to use, and document good practices. Huddles were characterized by a user-centered design approach, in which clinical and patient users would test features of the app during the prototype development phase and provide weekly feedback to the Genia design team who would modify the app based on this feedback. The huddles thus facilitated iterative feedforward or feedback problem-solving cycles that allowed for rapid identification of challenges to effective Genia use and rapid implementation of responsive action plans. For example, during one huddle, discomfort with using the technology was revealed as a potential barrier to use. The design team was able to quickly develop an instructional video, which clinical team members could view repeatedly to increase their proficiency with the app. In another example, the huddle discussions revealed that receiving previsit reports throughout the week was hard to manage within the clinical workflow. The team instead tried receiving previsit reports every Sunday night triggered by an auto-prompt sent via the app to patients over the weekend; this proved to be more clinically manageable and further helped to structure the Monday huddle meetings. The team’s commitment to weekly meetings ensured that progress continually moved forward.

It’s been very important to have weekly contact with Genia. To remind you to ‘Think Genia!’.

Codecision making during the huddles about Genia development, and a patient-centered approach in which the pediatric CF patients and the clinical providers were considered the “experts,” both fostered engagement by the primary end users and enhanced motivation for continued Genia use. Patient and family lead users offered new ideas for upcoming iterations, provided a platform for peer support, and operated as ambassadors for spread. Huddles also fostered motivation through sharing of successful patient or provider stories of Genia use and lessons learned. Thus, the huddles represented the primary mechanism by which Genia progressed from initial design and limited use to wider scale adoption.

### Engagement in Incremental “Genia Talk” Motivated Patient Adoption

The physiotherapists in Lund were primarily responsible for introducing Genia to patients. Several strategies for doing so were collectively referred to as “Genia Talk” by the physiotherapists. When possible, the physiotherapists introduced Genia to patients as early as possible in their care management. This communicated to the patient the integration of Genia with the usual care offered by the clinic.

The physiotherapists made Genia their tool. They signal very clearly to all patients that Genia is a way of working for them. This is how we do it in Lund.

Physiotherapists utilized a tailored, stepwise approach to the introduction of the app. Rather than showing patients (and their families) the entire app all at once, the physiotherapists selected one feature of the app to focus on with the patient at a particular visit (eg, how to record a daily observation), tailored to a current clinical need. At the next clinical visit, the physiotherapists would check on the progress with that feature of the app and introduce another new feature (eg, how to track medications).

We present Genia and what we can, together, benefit from in the app. We don’t try to present everything at once, but take small steps forward.

Additionally, at every visit, the physiotherapist would ask to collaboratively complete the previsit report in Genia with the patient if the patients had not completed the previsit reports on their own. This new process was initially tested by one physiotherapist as a response to patients forgetting to complete the previsit report prior to arriving at the clinic; due to its success in activating patients to use Genia and helping to shape their expectations about the importance of the report, this process was subsequently normalized within the clinical setting. This supported and gradual approach to onboarding patients was a central feature of the successful adoption of Genia by patients. By allowing patients to learn the app in a way that was responsive to their current needs, patients incrementally experienced the heightened value of Genia over time without increased burden. As more and more patients began using the app in a successful way, word-of-mouth spread patient interest in using Genia, which also contributed to a transition to wider usage.

### Co-Design Approach Toward Pediatric Cystic Fibrosis Care

Emerging from the weekly huddles and improved engagement with patients was a process characterized by co-design strategies, where conversations and interactions were iterative and collaborative. These co-design strategies were profiled by the Lund providers in contrast to previous practice strategies marked by one-sided conversations and asymmetric information sharing. The co-design strategy ensured that the app was viewed as relevant, usable, and valuable to both patients and clinicians, thus overcoming potential barriers to implementation during the design phase rather than waiting until after the app was completed. Thus, the coproduction of the app itself was inherent to the success of the integration process.

In Genia-supported CF care, patients were able to share challenges and insights prior to meeting with their care team, which permitted care team members to prepare and more efficiently and effectively interact with them. One clinician said, “Being prepared for the visit, both family and patient” was made possible by the previsit report function through Genia. The functionality of Genia also permitted the cocreation of care notes, which was an outcome driven by the previsit report function. The efficiency of the structured visit, and “knowing what to ask,” helped the clinicians focus on the treatment plans for their patients rather than on managing the visit itself.

## Discussion

### Principal Findings

This descriptive case study contributes to a small body of literature on technology-based interventions to improve CF self-management [25,26] and is one of the first to document the development and adoption process of an mHealth app for pediatric CF patients in a clinical setting. By illustrating successful features of integration, our findings highlight cultural characteristics of the clinical setting, which are more likely to support the feasible integration of new technologies, as well as mHealth design components that contribute to success.

This case reveals how the team-based institutional culture of CF care practice in Sweden complemented readiness for adoption of a novel mHealth patient engagement tool. Multiple characteristics of the clinic itself set the stage for successful integration, including cultural ethos of QI, desire for more modern ways of providing care, relational co-ordination within a small care team [27], and mHealth champions who represented the most influential clinical team members both within the team and with patients, and thus could foster shared aims. These characteristics, which some have referred to as an “implementation climate,” [28] essentially primed Lund for the successful adoption of Genia. Organizational readiness of the clinical setting thus plays a gate-keeping role in mHealth integration [29]. As such, it offers important insights into the selection of settings that are likely to successfully adopt mHealth technologies for pediatric chronic illness management. This approach, however, is a resource-intensive process. Cultural and contextual characteristics of many pediatric clinical settings may reduce their ability to meet weekly for QI huddles or to engage patients in the intensive mHealth design process.

Weekly team huddles during the adoption phase of Genia enhanced routinization of communication practices and the collective belief in the value of a technology-based application to engage CF patients. In essence, these weekly huddles served as the primary mechanism that operationalized the iterative, user-focused design process. Ample research on the development of mHealth technology promotes the incorporation of end user in the design process [30]. However, often this user input is incorporated late in the design phase or episodically. Our case demonstrates that weekly meetings between key stakeholders (ie, patients and clinicians) and the technology design team beginning early in the app development process were critical to integration. Not only did this human-centered design approach [30] allow for frequent, iterative design cycles based on user feedback (ie, feedforward or feedback information cycles), it also enhanced the commitment to use Genia due to a collective feeling that Genia is “for us, by us.”

The gradual, patient-specific onboarding process used by the physiotherapists enabled patients to learn to use Genia in an incremental manner. By highlighting features of the app that were useful to patients as needed, patients saw the increasing value of Genia without being overwhelmed by the capabilities of the technology. Since ease of use is one of the most frequently cited barriers to mHealth adoption [13,15], this slow and steady process of exposure, always with the direct help of the physiotherapist, was central to patients’ positive experiences with the app.

### Limitations

There are several limitations in this case study report. The sample of qualitative data was relatively small and was examined retrospectively, which limited our ability to pursue clarification of historical artifacts. Results presented here should be regarded as preliminary. Our case study examined only one clinical setting in Sweden, limiting the generalizability of the findings. Future work should examine the Genia adoption process at other pediatric CF centers to determine whether facilitators identified in this study are transferable to other clinical settings. Additionally, examining how care teams collaborated in their use of Genia with respect to specific care regimens—such as nutrition or physical activity—would be useful to reveal the microsystem-level utility of the app. We recognize that direct patient’s perspectives (eg, patient interviews) were not included in the data sources, as this case was focused on the implementation and dissemination of the technology within the Lund clinical setting and among its clinicians. Patient perspectives are necessary to directly examine how patients perceived the clinical integration of Genia in Lund. Our report may have been limited by the Swedish and English language challenges of communicating across cultures. Although the case report was developed in partnership with bilingual partners and many clinicians spoke both English and Swedish, the nuance of the details must be read with caution as translations and interpretations may vary. Finally, this study only examined the process of adoption and implementation of Genia and did not examine the impact of Genia on patient outcomes, such as improved self-management, treatment adherence, or quality of life. Future studies among patients are needed to determine whether Genia improves care compliance to and self-management of CF, and ultimately patient health outcomes. These offer important research directions for the use of Genia by pediatric CF patients.

### Conclusions

This qualitative case study offers preliminary evidence for strategies necessary for the successful adoption of an mHealth app within a pediatric chronic illness clinical setting. Although originally designed for pediatric patients with CF, the process described here could be applied to any pediatric chronic illness requiring extended self-management and complex care with multisector teams.

## Abbreviations

CF: cystic fibrosis
mHealth: mobile health
PSS: patient support system
QI: quality improvement
